# 

**Published:** 1983-07

**Authors:** 


JULY 1983

VOLUME 98 (iii)

NUMBER 367

Published Quarterly

Annual Subscription £8 Post Free

### BRISTOL MEDICO-CHIRURGICAL JOURNAL

Contents

97. **Editorial**

Established 1883

### BRISTOL MEDICO-CHIRURGICAL JOURNAL

Incorporating the Medical Journal of the South West

136. 100 Years Ago

139. From Our Correspondents

144. From Our Foreign Correspondent

148. Centenary Dinner for the Bristol Medico-Chirurgical Journal

149. South West Orthopaedic Club

152. Cossham Medical Society 1982-83

153. List of Members

Printed in Great Britain by John Wright & Sons (Printing) Ltd, at The Stonebridge Press, Bristol

### Editorial Committee

**EDITOR**
*Mr. M. G. Wilson*
*Litfield House, Clifton, Bristol BS8 3LS*

ASSISTANT EDITOR
*Dr. C. R. Tribe*
*Southmead Hospital, Bristol BS10 5NB*

MEMBERS
*Dr. M. B. Lennard*
*Dr. D. W. Wright*
*The Hon. Treasurer of the Society*
*The Hon. Secretary of the Society*

SECRETARY
*Mr. B. P. Jones, The Library, Medical School, University Walk, Bristol BS8 1TD*

CORRESPONDENTS
*Dr. Joseph Jancar*
*Dr. Gordon Mather*
*Professor Gordon Stirret*
*Dr. Gordon Thomson*
*Dr. Colin Tribe*
*Dr. Michael Whitfield*
*Professor Robin Williamson*
*Dr. Dennis Wright*

### Bristol Medico-Chirurgical Society

PRESIDENT 1982/83
*Mr. Herbert Bourns*

PRESIDENT ELECT
*Dr. Norman Tricks*

HONORARY SECRETARY
*Dr. H. M. Norman*
*Hillside, Vicarage Lane, Barrow Guerney*

HONORARY TREASURER
*Dr. J. Penry*
*Southmead Hospital, Bristol BS10 5NB*

The Society was founded in 1874. In 1883 publication of the Journal began and has continued quarterly ever since then. During the years 1949 to 1962 it appeared under the title of 'The Medical Journal of the South West'.

### Notice to Contributors

*The Journal is especially concerned to provide a place for the recording of medical thought, interests, and practice in and around Bristol. It therefore welcomes articles that, as well as being of immediate interest to members, will document the local and contemporary medical scene for our successors.*

*Original articles are invited provided that they have not been published elsewhere. Illustrations should be supplied ready for reproduction. Line drawings should be in Indian ink on firm white paper or board. The author will be informed if it is found necessary to ask him to pay for the printing of photographs, etc.*

*The following contributions will be especially welcome: notes of forthcoming events, meetings held, degrees conferred, staff appointments, honours and public appointments and other local news.*

*Advice on preparation can be obtained from the Editor.*

### BRISTOL MEDICO-CHIRURGICAL SOCIETY

### List of Members

Mr. T. M. Abbas, 9 Stoke Hill, Stoke Bishop, Bristol
Dr. Emily Adlam, Manor Farm, East Horrington, Wells, Somerset
Dr. G. R. Airth, Trevi House, Silverhill Brake, Rudgeway, Bristol
Mr. A. C. Akehurst, Castle Hill House, Nether Stowey, Bridgwater
Dr. B. C. E. Aldous, 40 Gloucester Road, North Bristol
Dr. John Aldren, 90 Pembroke Road, Clifton, Bristol
Professor R. J. Ancill, Yarley Hill Farm, Yarley, Wells, Somerset
Dr. J. H. Andrews, Lockstone, 58 Station Road, Shirehampton, Bristol
Dr. D. L. Archer, Penhill, 81 Station Road, Shirehampton, Bristol
Dr. K. B. Arney, Summerhazes, Cradley, Near Malvern, Worcestershire

Dr. A. Bailey, Stafford Lodge, Chesterfield Hospital, Clifton Hill, Bristol
Dr. I. S. Bailey, 17 Elmlea Avenue, Bristol
Dr. Jana S. Bailey, 7 Old Vicarage Green, Keynsham, Bristol
Dr. P. B. Bailey, Staunton Lodge, Staunton Lane, Whitchurch, Bristol
Mr. R. A. J. Bailey, 11 Elmhyrst Road, Weston-super-Mare, Avon
Mr. Roger N. Baird, 23 Old Sneed Park, Stoke Bishop, Bristol
Dr. Caroline J. Baker, 58 Pembroke Road, Clifton, Bristol
Dr. Hugh Bannerman, 3 New Street, Wells, Somerset
Dr. R. D. Barber, Geriatric Day Hospital, Frenchay Hospital, Bristol
Dr. R. F. Barbour, 7 Percival Road, Clifton, Bristol
Dr. M. G. Barker, Barrow Hospital, Barrow Gurney, Bristol
Dr. D. W. Barritt, Wycke House, 55 Long Ashton Road, Long Ashton, Bristol
Dr. P. J. F. Baskett, 21 West Mall, Clifton, Bristol
Dr. D. L. Bayley, 10 Tarnwell, Stanton Drew, Bristol
Dr. D. H. Beatson, 37 Beaufort Road, Clifton, Bristol
Dr. J. H. Beatson, The Gables, 4 Rayleigh Road, Bristol
Dr. A. L. T. Beddoe, 9 Pyecroft Avenue, Henleaze, Bristol
Dr. J. A. Bennett, Frenchay Hospital, Frenchay Park Road, Bristol
Dr. M. O. Bennett, 37 West View Road, Keynsham, Bristol
Dr. J. E. Beviss, 8 Queen's Parade, Bath, Avon
Dr. K. D. Bhoola, 24 Bramble Drive, Bristol
Mr. P. G. Bicknell, E.N.T. Department, Bristol General Hospital, Bristol
Dr. Joy S. Blight, Trymwell Cottage, Waters Lane, Westbury-on-Trym, Bristol
Dr. R. Blight, Trymwell Cottage, Waters Lane, Westbury-on-Trym, Bristol
Mr. D. C. Bodenham, 31A Old Sneed Road, Stoke Bishop, Bristol
Dr. Joy Bothamley, 3 Bell Barn Road, Stoke Bishop, Bristol
Mr. H. Bourns, 5 Downfield Road, Clifton, Bristol
Dr. John Bowes, Brook Farm House, Publow, Somerset
Dr. Joseph Boyle, Woodfield Cottage, Quarry Road, Frenchay Village, Bristol
Dr. E. C. Bradbeer, 28 Salterns Way, Lilliput, Parkstone, Dorset
Dr. B. Bradley, Whiteshill House, Whiteshill Common, Hambrook, Bristol
Dr. James C. Briggs, 194 Stoke Lane, Westbury-on-Trym, Bristol
Professor R. J. Brocklehurst, Cleeve, Court Road, Newton Ferrers, Plymouth, South Devon
Mr. C. A. Brown, 11 Southover Close, Westbury-on-Trym, Bristol
Dr. Jean C. Brown, Rock Farm, Longhope, Gloucestershire
Dr. N. J. Brown, The Old Vicarage, Portbury, Bristol
Dr. R. M. Brown, 36 Wick Crescent, Brislington, Bristol
Dr. J. I. Buckner, Park House, Avonmouth, Bristol
Professor A. J. Buller, Flat 8, Seawalls, Seawalls Road, Bristol
Dr. David Burman, 2 Chew Court Farm, Chew Magna, Bristol
Dr. W. H. Burnham, 27 Cleveland Close, Thornbury, Bristol
Dr. C. J. Burns-Cox, Department of Medicine, Frenchay Hospital, Bristol
Dr. G. W. Burton, Woodgrove, 35 Old Sneed Avenue, Stoke Bishop, Bristol
Dr. J. L. Burton, Poulton Lodge, Hazelwood Road, Sneyd Park, Bristol
Professor N. R. Butler, Children's Hospital, St. Michael's Hill, Bristol

Dr. D. Cameron, 1 Dean Lane, Southville, Bristol
Dr. M. J. Campbell, 20 Church Road, Sneyd Park, Bristol
Dr. J. P. Capel, Consultant in Charge, Accident and Orthopaedic Department, Frenchay Hospital, Bristol
Dr. Ian Capstick, St. Peter's Hospice, Tennis Road, Knowle, Bristol
Dr. F. Carswell, 12 Henleaze Gardens, Henleaze, Bristol
Dr. G. P. Carter, 42 Kensington Hill, Staple Hill, Bristol
Dr. Mary Carter, Yeobank, Congresbury, Bristol
Dr. J. E. Cates, 11 Cedar Park, Stoke Bishop, Bristol
Dr. Alison Causton, Wigmore House, Thornbury, Bristol
Dr. John Causton, Wigmore House, Thornbury, Bristol
Dr. H. R. Cayton, 19 The Drive, Bristol
Dr. E. Joanna Chambers, 4 Clyde Park, Bristol
Dr. T. L. Chambers, 4 Clyde Park, Bristol
Dr. W. A. W. Chapman, 2 St Oswald's Road, Bristol
Dr. I. D. Chisholm, Bourton House, Flax Bourton, Bristol
Professor J. R. Clamp, 26 Victoria Square, Bristol
Dr. Suzanne K. R. Clarke, Flat 3, Hambledon House, 20 Cotham Road, Bristol

Dr. Alan Clement, 9 Rylestone Grove, Stoke Bishop, Bristol
Mr. Hugh Coakham, Frenchay Hospital, Frenchay Park Road, Bristol
Dr. D. R. Coles, Department of Medicine, Bristol Royal Infirmary, Bristol
Professor J. R. T. Colley, Department of Community Health, Canynge Hall, Clifton, Bristol
Dr. Paul J. Conway, 20 Woodhill Road, Portishead, Bristol
Dr. N. Cook, 590 Wells Road, Bristol
Dr. Brian Cooper, Laurels, Acton Turville, Badminton, Avon
Dr. D. J. Cooper, 159 Bradford Road, Combe Down, Bath, Avon
Dr. Beryl Corner, 4 Elton House, Rodney Place, Clifton, Bristol
Dr. J. S. Cornes, 2 Westbury Park, Bristol
Dr. Jacqueline Cornish, 31 Wellington Park, Clifton, Bristol
Dr. J. C. Cornwell, 12 Knoll Hill, Sneyd Park, Bristol
Dr. R. J. Corrall, 64 Pembroke Road, Clifton, Bristol
Dr. J. A. Cosh, Roundoak House, 27 Croft Bank, W. Malvern, Worcestershire
Dr. Pamela Counsell, Flat 4, 8 Clifton Park, Bristol
Mr. J. Kevin Craig, Apthorp, Weston Road, Bath, Avon
Dr. R. A. Craig, 15 Old Sneed Park, Bristol
Dr. Aerona Crick, Kelvin House, Winscombe, Avon
Dr. M. Crick, Kelvin House, Winscombe, Avon
Dr. Macdonald Critchley, The National Hospital, Queen Square, London
Mr. J. Crossley, 9 Christchurch Road, Clifton, Bristol
Dr. H. J. Crow, Northwick Hall, Northwick Road, Pilning, Bristol
Dr. D. M. Cunningham, 23 Carnarvon Road, Redland, Bristol

Professor A. I. Darling, 7 Rylestone Grove, Stoke Bishop, Bristol
Dr. A. A. K. Datta, 19 Old Aust Road, Almondsbury, Bristol
Dr. Timothy J. David, 6 Colwyn Avenue, Alkrington, Middleton, Manchester
Mr. C. M. Davidson, 69 Coombe Lane, Bristol
Professor E. Rhys Davies, 22 Blenheim Road, Westbury Park, Bristol
Dr. Hugh G. Davies, Old Kingshill Farm, Nailsea, Bristol
Dr. J. D. Davies, 20 Clifton Park, Clifton, Bristol
Professor D. Russell Davis, 9 Clyde Road, Redland, Bristol
Dr. D. McD. Dickson, Oldlands, Frenchay Common, Bristol
Dr. Margot Dickson, 9 Mortimer Road, Clifton, Bristol
Dr. Janet H. Dix, 3 Stanishaw Close, Frenchay, Bristol
Dr. W. E. Dobson-Smyth, 128 High Street, Hanham, Bristol
Dr. I. L. Donaldson, 86 Berkeley Road, Bristol
Dr. A. Dowling, Stafford House, 1 All Saints Road, Clifton, Bristol
Dr. A. A. Dowling, 87 Parry's Lane, Bristol
Dr. W. J. P. Duff, 4 Victoria Square, Clifton, Bristol
Mr. J. C. Du Heaume, 10 Water Lane, Pill, Bristol
Dr. Andrew Duncan, Bristol Children's Hospital, St. Michael's Hill, Bristol
Dr. B. F. Dunleavy, 29 Oldbury Court Road, Fishponds, Bristol
Dr. P. Dunn, Tramore, Henbury Road, Henbury, Bristol
Dr. I. A. R. Dunnett, Fordbank Cottage, Yate Rocks, near Chipping Sodbury

Dr. D. F. Early, Glenside Hospital, Blackberry Hill, Stapleton, Bristol
Dr. Z. Eastes, Moons Close, Sheep Fair Lane, Marshfield, Chippenham, Wiltshire
Dr. F. V. Eastman, 37 Shirehampton Road, Bristol
Dr. R. D. Eastman, Frenchay Hospital, Bristol
Professor David L. Easty, Litfield House, Clifton, Bristol
Dr. H. Eckert, 36 Church Road, Bristol
Dr. A. C. Edwards, Brantfell, Woollard Lane, Whitchurch, Bristol
Dr. Nigel Edwards, Litfield House, 1 Litfield Place, Clifton, Bristol
Dr. Janet Elphick, 2 St. Margarets Close, Backwell, Bristol
Mr. W. K. Eltringham, 4 Bucklands Grove, Nailsea, Avon
Professor M. A. Epstein, Department of Pathology, Medical School, University Walk, Bristol
Dr. R. Esler, The Little Mythe, Julian Road, Sneyd Park, Bristol
Mr. H. J. Espiner, 21 Woodland Grove, Bristol
Dr. Jean Eustace, 47 Englishcombe Lane, Bath, Avon
Dr. B. R. W. Evans, Bibury, Old West Town Lane, Brislington, Bristol
Dr. David Evans, 39 Boulevard, Weston-super-Mare, Avon
Dr. G. M. Evans, 117 Ashley Road, Bristol
Dr. R. F. Everton, 46 Capel Road, Lawrence Weston, Bristol
Mr. A. L. Eyre-Brook and Dr. Edith M. Eyre-Brook, 8 Lodge Drive, Long Ashton, Bristol

Mr. H. D. Fairman, 9 Clifton Park, Clifton, Bristol
Mr. D. Faulkner, 54 Russell Grove, Westbury Park, Bristol
Dr. P. J. Featherstone, 10 West Mall, Clifton, Bristol
Dr. G. N. Febry, 11 Salisbury Road, Redland, Bristol
Dr. G. L. Feneley, Avon Lodge, 8 Parry's Lane, Bristol
Mr. R. C. L. Feneley, 51 Canynge Road, Clifton, Bristol
Dr. R. H. P. Fernandez, Senior Medical Officer, Central Electricity Generating Board, Bridgwater Road, Bristol
Dr. J. G. Field, The Three Jays, Kilcott Road, Hillesley, Wotton under Edge, Gloucestershire
Dr. Shelagh M. Fitzgibbon, Ladybrook Cottage, Laverton, near Broadway, Worcestershire
Dr. Alec Forbes, 19 Druid Woods, Avon Way, Stoke Bishop, Bristol
Dr. R. Y. Forbes, 6 Oakfield Mansions, Oakfield Grove, Bristol
Dr. G. L. Foss, 3 Litfield Place, Clifton, Bristol
Mr. J. E. B. Foulkes, 83 Priory Road, Compton Village, Plymouth, South Devon
Dr. D. B. Fowler, The Red Lodge, Abbots Leigh Road, Leigh Woods, Bristol
Dr. D. H. Fox, Harts, Gloucester Road, Woodhouse Down, Almondsbury, Bristol
Dr. C. J. Fraser, Cornerways, Dragons Well Road, Brentry, Bristol
Dr. I. D. Fraser, 18 Owen Grove, Henleaze, Bristol

Dr. S. E. Fraser, Cornerways, Dragons Well Road, Brentry, Bristol
Dr. Marie J. Freeman, Pound Cottage, Jubilee Lane, Cromhall, near Wotton under Edge, Gloucestershire

Dr. H. D. T. Gawn, 270 Wells Road, Bristol
Dr. F. R. Gedye, Huntercombe, 4B Westbury Lane, Bristol
Dr. M. J. Gibson (present address unknown)
Professor W. A. Gillespie, 2 Church Avenue, Stoke Bishop, Bristol
Dr. John H. Gillmore, 5 Bucklands Batch, Nailsea, Bristol
Mr. J. Clive Gingell, 15 The Crescent, Henleaze, Bristol
Dr. A. T. M. Glen, 139 Court Farm Road, Whitchurch, Bristol
Dr. S. C. Glover, Ham Green Hospital, Pill, Bristol
Dr. D. J. Goldie, Department of Clinical Chemistry, Southmead Hospital, Bristol
Dr. R. P. Golding, Corbett House, Avonvale Road, Bristol
Dr. N. H. H. Golledge, The Court, Axbridge, Somerset
Dr. J. B. Gordon-Russell, Hortham Hospital, Almondsbury, Bristol
Dr. Clive Grattan, Department of Dermatology, Bristol Royal Infirmary, Bristol
Dr. Susan R. Greenwood, Timbertops, Cadbury Camp Lane, Clapton-in-Gordano, Bristol
Dr. Pat M. Grenfell, 5 Druid Road, Bristol
Mr. Huw B. Griffith, Department of Neurosurgery, Frenchay Hospital, Bristol
Mr. Mansell Griffiths, 2 Clifton Park, Clifton, Bristol
Dr. Jane Grubb, Garden Flat, 2 College Road, Clifton, Bristol

Dr. Brendan T. Hale, Radiotherapy Department, Bristol General Hospital, Bristol

Dr. M. E. H. Halford, Fourstacks, 18 Druid Stoke Avenue, Stoke Bishop, Bristol
Dr. Elizabeth W. Hall, 6 Claremont Place, Bath, Avon
Dr. K. A. Hall, 52 Clifton Down Road, Bristol
Dr. E. P. Hamblett, 93 Gloucester Road North, Filton, Bristol
Dr. W. L. Hardman, 20 Dial Hill Road, Clevedon, Avon
Dr. Jane Hargreaves, Woodview House, Tideford, near Saltash, Cornwall
Dr. R. R. M. Harman, Department of Dermatology, Phase I Building, Bristol Royal Infirmary, Bristol
Dr. J. E. Harrison, 43 Gloucester Road North, Bristol
Dr. Joy Harrison, Pathology Department, Frenchay Hospital, Bristol
Dr. Peter B. Harrison, 48 Russell Grove, Bristol
Mr. J. C. Dean Hart, 97 Long Ashton Road, Long Ashton, Bristol
Dr. G. Hartill, Stone House, Chipping Sodbury, Gloucestershire
Dr. M. Hartog, Department of Medicine, Bristol Royal Infirmary, Bristol
Dr. R. F. Harvey, 25 Shipley Road, Westbury-on-Trym, Bristol
Dr. R. V. Havard, 2 Tichborne Road, Weston-super-Mare, Avon
Dr. I. A. Hawkins, Brooklands, Easton-in-Gordano, Bristol
Dr. J. M. Haworth, 72 Bifield Road, Stockwood, Bristol

Dr. K. W. Heaton, Claverham House, Stream Cross, Claverham, Bristol
Dr. W. A. Heaton Ward, 75 Pembroke Road, Clifton, Bristol
Dr. G. D. Henley, 58 Pembroke Road, Clifton, Bristol
Dr. G. C. K. Herepath, 12 Beech Road, Saltford, Bristol
Dr. A. G. Heron, Woodrange, 26 Henbury Road, Westbury-on-Trym, Bristol
Dr. R. Langton Hewer, Three Gables, Valley Road, Leigh Woods, Bristol
Professor T. F. Hewer, Vine House, Henbury, Bristol
Dr. G. M. Higgins, 71A Parry's Lane, Bristol
Mr. R. W. Hiles, Up Yonder, Moorend, Hambrook, Bristol
Dr. P. G. Hill, 45 Stoke Hill, Stoke Bishop, Bristol
Dr. J. M. Hoffman, 5 Abbey Road, Westbury-on-Trym, Bristol
Dr. N. J. Hooper, Langford Place, Langford, Bristol
Mr. Michael Horrocks, 30 Park Crescent, Frenchay, Bristol
Dr. W. Hughes, The Green, Wick, Bristol
Dr. J. S. Hughes-Games, William Budd Health Centre, Leinster Avenue, Knowle, Bristol
Mr. Michael Hull, Bristol Maternity Hospital, Southwell Street, Bristol
Mr. A. Hulme, Department of Neurosurgery, Frenchay Hospital, Bristol
Dr. R. J. Hunt, Pen-y-Banc, Pensford, Bristol

Dr. N. B. N. Ibrahim, Consultant Histopathologist, Frenchay Hospital, Bristol
Dr. A. H. Iles, 1 Pembroke Vale, Clifton, Bristol
Dr. R. A. Iles, 7 Hurle Crescent, Bristol

Dr. A. G. Jackson, 54 Fallodon Way, Henleaze, Bristol
Dr. D. H. James, Hilbre, 1 Grove Road, Combe Dingle, Bristol
Mr. J. A. James, Litfield House, Clifton Down, Bristol
Dr. J. Jancar, Emona, Beaufort Place, Frenchay Manor Park, Bristol
Mr. P. Jardine, Bristol Eye Hospital, Bristol
Dr. W. D. Jeans, 100 Redland Road, Bristol
Dr. D. C. Jeffery, 24 Monks Park Avenue, Horfield, Bristol
Mr. K. Jeyasingham, Frenchay Hospital, Frenchay Park Road, Bristol
Mr. A. H. John, 5 Upper Belgrave Road, Clifton, Bristol
Dr. Derek H. Johnson, 4 Alexandra Road, Clifton, Bristol
Mr. D. M. Jones, 3 Greenland Terrace, Aberaeron, Dyfed, Wales
Dr. M. Cemlyn Jones, 2 Kensington Place, Clifton, Bristol
Dr. S. C. Jordan, 5 Leigh Road, Bristol
Dr. Mary C. A. Jorro (Mrs. M. Cantwell), Birchwood, Park Lane, Ystrad Mynach, Hengoed, Glamorgan
Dr. Robin Joy, Harbells, Ropers Lane, Wrington, Bristol
Dr. Vyvyan J. Joy, Harbells, Ropers Lane, Wrington, Bristol
Dr. David N. Joyce, Department of Obstetrics, Southmead Hospital, Bristol

Mr. Gerald Keen, Frenchay Hospital, Bristol
Dr. T. D. Kellock, Edbrook House, 1 Old Town, Wotton under Edge, Gloucestershire
Dr. W. P. Kelly, 7 Woodland Road, Weston-super-Mare, Avon

Dr. C. T. C. Kennedy, 1 Cornwallis Crescent, Clifton, Bristol
Dr. A. M. Kingon, Gatcombe, Winscombe, Avon
Dr. J. D. Kirby, Summerleaze, East Harptree, Bristol
Mr. John Kirkup, Royal United Hospital, Bath, Avon

Dr. E. J. Lace, 15 Julian Road, Bristol
Dr. E. M. Lace, 15 Julian Road, Bristol
Dr. Evelyn M. Lafferty, Banks House, Winterbourne Down, Bristol
Dr. S. K. Lahiri, Drylaw Cottage, Cote Drive, Bristol
Dr. Campbell C. Laird, Lake House, Leigh Woods, Bristol
Dr. Robin G. Lambert, Bracken Cottage, 45 Silver Street, Nailsea, Bristol
Dr. R. D. Last, Coultings, Housman Road, Street, Somerset
Dr. G. Laszlo, Bristol Royal Infirmary, Bristol
Dr. A. S. Lavelle, 11 Dean Lane, Bedminster, Bristol
Dr. Richard H. Lawson, 10 Algiers Street, Bedminster, Bristol
Mr. David Leaper, Department of Surgery, Bristol Royal Infirmary, Bristol
Dr. G. E. P. Lee, 56 Pembroke Road, Clifton, Bristol
Dr. A. Leitch, 54 Hill Grove, Henleaze, Bristol
Dr. Lois M. Leitch, 3 Lodge Road, Caerleon, Gwent
Dr. Joan Lennard, 9 Chapel Green Lane, Redland, Bristol
Dr. Michael Lennard, 9 Chapel Green Lane, Redland, Bristol
Professor G. Gordon Lennon, Mediaeval House, The Square, Axbridge, Somerset
Dr. J. V. Lever, 23 Penn Lea Road, Bath, Avon
Dr. F. J. W. Lewis, Osborne House, Fallodon Way, Henleaze, Bristol
Dr. Sylvia Lewis, 2 Downs Cote View, Westbury-on-Trym, Bristol
Dr. E. Little, 29 Apsley Road, Clifton, Bristol
Dr. O. C. Lloyd, Withey House, Withey Close West, Bristol
Dr. W. H. Lloyd, Manor Park Hospital, Fishponds, Bristol
Dr. Ian A. Lowe, Beresford House, 1 Clifton Hill, Bristol
Dr. G. Lucas, 51 Sea Mills Lane, Bristol
Dr. G. N. Lumb, Highfield, Hope Corner Lane, Taunton, Somerset
Dr. B. S. Lush, Frenchay Hospital, Bristol
Dr. Margaret Lush, Witton House, The Quarries, Almondsbury, Bristol
Dr. Lucy M. Lynch, 12 The Willows, Frenchay Manor Park, Bristol
Dr. Anne F. M. Lyons, 72 Birchwood Road, Bristol

Dr. A. W. Macara, Department of Community Health, Canynge Hall, Whiteladies Road, Bristol
Dr. D. McCarthy, 12 Cote Park, Stoke Park, Bristol
Mr. M. P. McCormack, 18 Richmond Hill, Bristol
Dr. D. R. McCoy, 34 Canynge Square, Clifton, Bristol
Dr. A. J. R. Macdonald, 19 Richmond Hill, Bristol
Dr. A. D. McInnes, c/o General Hospital, St. Helier, Jersey, Channel Islands
Dr. J. C. Mackenzie, Southmead Hospital, Bristol
Dr. A. N. McLean, Bristol Royal Infirmary, Bristol
Dr. A. I. MacLeod, 35 Dial Hill Road, Clevedon, Avon
Dr. J. M. McLeod, 17 Woodhill Road, Portishead, Bristol
Dr. I. A. McQuade, 20 Queen's Road, Bishopsworth, Bristol
Dr. J. Macrae, Upland Cottage, Tower House Lane, Wraxall, Avon
Mr. Richard Markham, 86 Pembroke Road, Clifton, Bristol
Mr. V. J. Marmion, 73 Pembroke Road, Clifton, Bristol
Dr. Phillipa Marsh, Belvedere, North Road, Leigh Woods, Bristol
Dr. M. S. Marston, Woodpeckers, Warren Lane, Long Ashton, Bristol
Dr. J. P. Martin, 1 Lodge Drive, Long Ashton, Bristol
Dr. H. G. Mather, 10 Avon Grove, Bristol
Dr. C. N. A. Matthews, 8 Carnarvon Road, Bristol
Mr. A. R. Maw, Litfield House, Litfield Place, Bristol
Dr. R. B. H. Maxwell, Quarry House, Charlton Road, Westbury-on-Trym, Bristol
Dr. A. J. Mayes, 19 Hartington Park, Bristol
Dr. Gwyneth Meadows, 14 Redland Court Road, Bristol
Dr. I. H. Mian, Glenside Hospital, Blackberry Hill, Stapleton, Bristol
Dr. Audrey Midwinter, St. Ives Farm, Doynton, Bristol
Dr. R. E. Midwinter, St. Ives Farm, Doynton, Bristol
Dr. J. Miles-Thomas, Ground Floor Flat, 10 Hope Square, Hotwells, Bristol
Dr. K. T. W. Miller, 15 West Town Road, Backwell, Somerset
Dr. C. A. Milner, 48 Bristol Road, Frenchay, Bristol
Mr. J. P. Mitchell, 23 Parry's Close, Bristol
Dr. Wendy Mitchell, 397 Eastfield Road, Westbury-on-Trym, Bristol
Dr. Norman Moore, 44 Stoke Lane, Westbury-on-Trym, Bristol
Dr. F. Morgan, Four Stacks, 23 Kingsweston Road, Henbury, Bristol
Dr. G. L. Morgan, Garden House, Stafford Place, Weston-super-Mare, Avon
Professor H. G. Morgan, 3 Downleaze, Stoke Bishop, Bristol
Dr. C. C. Morgans, 2 Long Acres Close, Bristol
Dr. T. Morley, 12 Owen Grove, Henleaze, Bristol
Dr. R. W. Morris, 36 High Street, Thornbury, Bristol
Dr. Martin Mott, 50 Downs Park West, Westbury Park, Bristol
Dr. K. W. Moulding, 18 St. Helen's Drive, Oldland, Bristol

Mrs. P. M. Neale, Ground Floor Flat, 3 Miles Road, Clifton, Bristol
Dr. A. S. Nethercott, Caledon House, Marton, Welshpool, Powys
Dr. Penelope Neville, 11 Southfield Road, Westbury-on-Trym, Bristol
Dr. Elizabeth M. Nicholson, Woodpeckers, Warren Lane, Long Ashton, Bristol
Mr. P. A. R. Niven, 21 Clifton Park, Bristol
Dr. H. M. Norman, Hillside, Vicarage Lane, Barrow Gurney, Bristol
Dr. Elizabeth M. Norris, Old Stables, Shrubbery Cottages, Redland, Bristol

Mr. M. O'Driscoll, Orthopaedic Department, Bristol Royal Infirmary, Bristol
Dr. D. E. Olliff, Grevel's House, Chipping Campden, Gloucestershire
Dr. H. J. Orr Ewing, 33 Worcester Court, Worcester Road, Clifton, Bristol

Dr. A. B. Otlet, 31 Oakfield Road, Clifton, Bristol
Dr. R. H. Owen, Coomesbury, 53 Loop Road, Beachley, Chepstow, Gwent

Dr. F. T. Page, Henbury Lodge, Station Road, Henbury, Bristol
Dr. K. Page, Fairwater, Dumpers Lane, Chew Magna, Bristol
Dr. T. F. Paine, 13 Limerick Road, Redland, Bristol
Mr. A. G. Palin, Springfort Down, Saville Road, Stoke Bishop, Bristol
Dr. J. L. Pardoe, Church Cottage, Church Road North, Portishead, Bristol
Dr. R. B. Pardoe, Tor House, Nore Road, Portishead, Bristol
Dr. A. S. Parker, 7 Priory Walk, Portbury, Bristol
Dr. R. D. G. Peachey, Department of Dermatology, Phase I Building, Bristol Royal Infirmary, Bristol
Professor J. H. Peacock, The Old Manor, Ubley, Bristol
Dr. Valerie Peet, 111 Pembroke Road, Clifton, Bristol
Dr. Charles Pennock, Pathology Department, Bristol Maternity Hospital, Southwell Street, Bristol
Dr. J. B. Penry, 20 Westbury Lane, Bristol
Mr. Barry Pentlow, Department of Surgery, Southmead Hospital, Bristol
Professor C. Bruce Perry, Beechfield, 54 Grove Road, Bristol
Dr. A. R. Peters, William Budd Health Centre, Leinster Avenue, Knowle West, Bristol
Dr. W. R. Phillips, 239 Cranbrook Road, Redland, Bristol
Mr. R. W. Pigott, Burlingham House, Bradley Road, Wotton-under-Edge, Gloucestershire
Dr. D. V. Pleasant, 12 Lodge Drive, Long Ashton, Bristol
Dr. G. E. J. Porter, 16 Princes Road, Clevedon, Avon
Dr. M. F. Potter, The Chalet, Grants Lane, Wedmore, Somerset
Dr. John N. Powell, Glen Elms, 17 Gloucester Road, Almondsbury, Bristol
Dr. P. A. Primrose, 25 West Town Lane, Brislington, Bristol
Dr. D. Pugh, Dunedin Lodge, Weston Road, Bath, Avon
Mr. R. Pyke, Bristol General Hospital, Bristol

Dr. D. S. Ractliffe, 22 St. Hilary Close, Stoke Bishop, Bristol
Dr. A. P. Radford, Crossways Cottage, West Bagborough, Taunton, Somerset
Dr. R. C. Raffety, 2 Harford Square, Chew Magna, Bristol
Mr. A. H. C. Ratliff, Bristol Royal Infirmary, Bristol
Professor A. E. A. Read, Department of Medicine, Bristol Royal Infirmary, Bristol
Dr. J. Russell Rees, Bristol General Hospital, Bristol
Dr. D. S. Reeves, 109 Long Ashton Road, Long Ashton, Bristol
Dr. T. D. Richards, Harwood Farm, Castle Road, Pucklechurch, Bristol
Dr. A. T. M. Roberts, 8 Harley Place, Clifton Down, Bristol
Professor C. Prys Roberts, Foxes Mead, Cleeve Hill Road, Bristol
Dr. J. A. Fraser Roberts, Paediatric Research Unit, Cameron House, Guy's Hospital Medical School, London
Dr. J. B. M. Roberts, 'Dormers', Woodlands Road, Portishead, Bristol
Mr. P. Hughes Roberts, Martindale, Bridgwater Road, Winscombe, Avon
Dr. K. P. Roche, White Lodge, Radnor Road, Westbury-on-Trym, Bristol
Mr. R. K. Roddie, 7 Percival Road, Clifton, Bristol
Dr. F. G. M. Ross, 2 Briercliffe Road, Coombe Dingle, Bristol
Mr. R. T. Routledge, Frenchay Hospital, Bristol
Dr. A. J. Rowland, 144 Stoke Lane, Bristol
Dr. John Roylance, 1 Wyck Beck Road, Brentry, Bristol

Dr. W. Ross Sadler, 70 Coombe Lane, Westbury-on-Trym, Bristol
Dr. N. G. Sanerkin, Bristol Bone Tumour Registry, Bristol Royal Infirmary, Bristol
Dr. E. Saphier, 20 Druid Woods, Avon Way, Stoke Bishop, Bristol
Mr. P. W. Sargeant, Hewbridge House, Lynn Road, Snettisham, Kings Lynn, Norfolk
Dr. D. C. L. Savage, Childrens Hospital, Bristol
Mr. Philip E. Savage, 34 The Crescent, Henleaze, Bristol
Dr. R. W. Sawford, 521 Filton Avenue, Northville, Bristol
Dr. Werner Schutt, 5 Grove Avenue, Coombe Dingle, Bristol
Dr. G. L. Scott, Quakers Meet, Kingsweston Road, Henbury, Bristol
Dr. M. P. Y. Scott, The Westra, 34A Church Road, Stoke Bishop, Bristol
Dr. Angela Shepherd, Pine Cottage, Old Down, Tockington, Bristol
Dr. D. H. Short, 3 Mariners Close, Backwell, Bristol
Dr. Hugh Silvey, 11 Park Hill, Shirehampton, Bristol
Dr. S. J. Silvey, 16 Westbury Lane, Bristol
Dr. M. M. Simmonds, 11 Dean Lane, Bedminster, Bristol
Dr. D. Simpson, 14 Shipley Road, Westbury-on-Trym, Bristol
Dr. P. J. Simpson, 2 St. Hilary Close, Stoke Bishop, Bristol
Norman Slade, Esq., Church Farm, Lower Failand, Bristol
Dr. J. A. Sluglett, 3A Alexandra Road, Clifton, Bristol
Dr. A. K. Smeeton, 46 Northumberland Road, Redland, Bristol
Dr. G. F. Smith, 118 Redland Road, Redland, Bristol
Dr. J. Spencer Smith, 28 Druid Stoke Avenue, Stoke Bishop, Bristol
Dr. K. C. P. Smith, 14 Howard Road, Redland, Bristol
Mr. P. J. B. Smith, Department of Urology, Bristol Royal Infirmary, Bristol
Mr. H. J. Drew Smythe, 2 Charlton Close, Charlton Kings, Cheltenham, Gloucestershire
Dr. A. H. Snaith, Avon Area Health Authority (T), Greyfriars, Lewins Mead, Bristol
Mr. W. F. W. Southwood, Upton House, Bathwick Hill, Bath, Avon
Mr. P. G. Stableforth, Bristol Royal Infirmary, Bristol
Dr. R. M. Stanford, 40 Kewstoke Road, Bristol
Dr. T. R. Steen, 21 Elgin Park, Redland, Bristol
Dr. M. P. Sternberg, 2 Clifton Park, Bristol
Dr. D. Stevens, 27 Granby Hill, Clifton, Bristol
Dr. David M. Stevens, 18 The Circus, Bath, Avon
Professor G. Stirrat, University Department of Obstetrics, Bristol Maternity Hospital, Bristol

Dr. D. W. G. Stone, 11 Dean Lane, Bedminster, Bristol
Dr. S. N. Stotesbury, Three Gables, Malmains Drive, Frenchay, Bristol

Mr. Norman C. Tanner, 5 Beaufort Road, Clifton, Bristol
Dr. E. A. Tarleton, 493 Long Cross, Lawrence Weston, Bristol
Dr. A. W. Taylor, 59 Calcott Road, Bristol
Dr. G. D. Teague, 210 Queen's Road, Withywood, Bristol
Dr. C. W. Thomas, 5 Grange Park, Westbury-on-Trym, Bristol
Mr. M. M. Thompson, 12 Avon Grove, Stoke Bishop, Bristol
Mr. Michael R. Thompson, 50 Clifton Park Road, Clifton, Bristol
Dr. P. D. Thompson, 7 Park View, Chepstow, Gwent
Dr. J. L. G. Thomson, Little Begbrook, Begbrook Park, Frenchay, Bristol
Dr. B. Tierney, The Orchard, Norton Lane, Chew Magna, Bristol
Mr. Michael Torrens, The Yews, Harry Stoke, Stoke Gifford, Bristol
Dr. K. M. Townend, Heywood, Pill, Bristol
Dr. C. R. Tribe, 10 Hyland Grove, Westbury-on-Trym, Bristol
Dr. N. C. Tricks, The Mill, Wrington, Bristol
Mr. C. Grant Tulloh, 33 Old Sneed Park, Stoke Bishop, Bristol
Dr. Gillian M. Turner, Department of Obstetrics and Gynaecology, Bristol Maternity Hospital, Bristol
Dr. R. C. Tyldesley, 267 Soundwell Road, Kingswood, Bristol

Dr. J. Virgee, 4 Salisbury Road, Redland, Bristol

Dr. G. S. Wakefield, Apthorp, Weston Road, Bath, Avon
Dr. W. Lumsden Walker, 6 Kellaway Crescent, Westbury-on-Trym, Bristol
Professor R. Milnes Walker, Wergs Copse, Kintbury, Newbury, Berkshire
Dr. R. V. Walley, Yew Tree House, Abbots Leigh, Bristol
Dr. T. B. Wallington, Amesbury House, 18 Gloucester Road, Almondsbury, Bristol
Dr. E. M. Walsh, 7 Burlington Road, Redland, Bristol
Dr. Ruth M. Walters, 10 Downs Road, Westbury-on-Trym, Bristol
Dr. Charles Ward, 7 Trelawney Road, Cotham, Bristol
Dr. R. P. Warin, Litfield House, Clifton Down, Bristol
Dr. S. A. F. Warren, 9 Alexandra Road, Clifton, Bristol
Dr. G. C. Watt, 1A Old Priory Road, Easton-in-Gordano, Bristol
Mr. A. John Webb, 10 Grange Park, Westbury-on-Trym, Bristol
Dr. Donald A. Webb, 1 Waterdale Close, Westbury-on-Trym, Bristol
Dr. M. A. H. Webb, Industrial Medical Adviser, Commonwealth Smelting Ltd., St. Andrew's Road, Avonmouth, Bristol
Dr. Hebe F. Welbourn, 44 Church Road, Winterbourne Down, Bristol
Dr. S. D. V. Weller, 2 Ormonde House, Sion Hill, Bath, Avon
Dr. R. West, The Old Rectory, Rowberrow, Winscombe, Avon
Dr. M. J. Whallett, Fairfield House, 58 Castle Street, Thornbury, Bristol
Dr. J. D. Wisheart, Department of Cardiac Surgery, Bristol Royal Infirmary, Bristol
Mr. H. J. O. White, Sundown, 22 Over Lane, Almondsbury, Bristol
Dr. R. J. White, Department of Medicine, Frenchay Hospital, Bristol
Dr. M. J. Whitfield, 24 Hanbury Road, Bristol
Dr. Peter Wilde, 49 Effingham Road, St. Andrew's Park, Bristol
Dr. D. J. Wiley, Sequoia House, Rownham Hill, Leigh Woods, Bristol
Dr. J. M. Wilks, The Lawn, Wick, near Bristol
Dr. E. W. Williams, 34 High Kingsdown, Bristol
Professor R. C. N. Williamson, University Department of Surgery, Bristol Royal Infirmary, Bristol
Mr. M. G. Wilson, The Red House, 40 Coombe Lane, Westbury-on-Trym, Bristol
Mr. P. J. Witherow, 9 Cooks Folly Road, Sneyd Park, Bristol
Dr. Grace Wood, 22 Westfield Road, Westbury-on-Trym, Bristol
Dr. A. W. Woolley, South Hill, Dulcote, Wells, Somerset
Dr. D. W. Wright, Ham Green Hospital, Pill, Bristol
Dr. H. J. Wright, Woodlands, Silver Street, Nailsea, Bristol
Dr. J. C. Wright, 22 Briercliffe Road, Westbury-on-Trym, Bristol

Dr. Derek W. Zutshi, 36 Eton Court, Eton Avenue, Hampstead, London

